# Quantifying the role of pre-existing tissue resident cellular immunity in limiting respiratory virus transmission

**DOI:** 10.1371/journal.ppat.1014082

**Published:** 2026-04-21

**Authors:** Ananya Saha, Sarah E. Michalets, Ida Uddbäck, Hasan Ahmed, Jacob E. Kohlmeier, Rustom Antia, Katia Koelle

**Affiliations:** 1 Department of Biology, Emory University, Atlanta, Georgia, United States of America; 2 Department of Microbiology and Immunology, Emory University School of Medicine, Atlanta, Georgia, United States of America; 3 Emory Center of Excellence for Influenza Research and Response (CEIRR), Atlanta, Georgia, United States of America; Thomas Jefferson University, UNITED STATES OF AMERICA

## Abstract

Viral transmission from infected donors to uninfected recipients is the key event underlying the spread of viral pathogens at the level of a host population. Successful viral transmission from a donor to a recipient depends on several factors including the infectiousness of the donor. Donor infectiousness in turn can depend on the viral kinetics and viral load of the donor, donor behavior and symptoms, and donor immunity. Here, we use a mouse model of murine respirovirus (otherwise known as Sendai virus SeV) infection to quantitatively explore donor determinants of respiratory virus transmission. The experimental transmission studies we analyze are specifically designed to address the effect that pre-existing donor immunity may have on transmission potential by studying SeV transmission from both immunized and control (placebo-immunized) donors to naïve recipients. We specifically focus on the impact of tissue resident memory (TRM) CD8 T cells on donor transmission potential by considering immunization strategies that primarily generate CD8 T cell immunity. Through quantitative analyses of these experiments, we find that pre-existing CD8 TRMs act to reduce donor transmission potential. This finding is in agreement with previous findings and can be in part explained by a reduction in total infection load in immunized donors. However, even once differences in infection load between immunized and control donors are accounted for, immunized donors still have reduced infectiousness relative to control donors. We explore possible reasons for this unexpected pattern using a mathematical model that integrates within-host viral dynamics and between-host transmission occurrences. Analysis of model simulations, along with observations from knock-out experiments, suggests that interferon gamma (IFN-γ) may be partly responsible for the observed differences in infectiousness between control and immune donors. Future experimental transmission studies should consider measuring IFN-γ levels and its effects when interpreting transmission outcomes in the context of host immunity.

## Author summary

Infection with respiratory viruses is a leading cause of illness and public health burden worldwide. Once someone becomes infected, blocking the transmission of the virus from infected donors to uninfected recipients is key to reduce the spread of these viruses in the population. In this study, we use a mouse model infected with Sendai Virus (SeV, a virus similar to human respiratory viruses) to explore how pre-existing immunity helps limit its transmission. We specifically focus on quantifying the role of tissue resident memory (TRM) CD8 T cells in blocking SeV transmission following infection. Our analyses focus on transmission from donor animals immunized with either a T cell vaccine generating CD8 TRM cells (immune donors) or a placebo (control donors). Following SeV infection of these control and immune donors, naïve recipients were exposed to infected donors for a short and fixed contact duration. We found that immune donors did not transmit the virus to naïve recipients, as opposed to control donors that showed robust transmission. We further considered data from another study where the contact duration between infected donors and naïve recipients was long and variable. In that study, immune donors stopped SeV transmission earlier than the control donors. By analyzing data from these two experimental transmission studies, we found that CD8 TRM cells limit transmission of SeV from infected immune donors by reducing their infection burden. Additionally, independent of infection burden, these cells reduce the propensity to transmit infectious virus per unit infection burden of immune donors. These results have implications on developing T cell-based vaccines to limit respiratory virus transmission.

## Introduction

Substantial transmission heterogeneity, or superspreading, underlies the dynamics of many infectious pathogens, such that the majority of secondary infections often stem from a small proportion of infected individuals [1,2]. Transmission heterogeneity is particularly well documented in respiratory viruses such as SARS-CoV-2 [3–8]. Understanding the sources of this heterogeneity is key to the design of effective intervention strategies. Many factors are known to contribute to transmission heterogeneity, including differences in host contact rates and in host infectiousness. Differences in host infectiousness are driven by differences in within-host viral kinetics as well as differences in disease symptoms such as coughing that can facilitate transmission [9,10]. In turn, host factors such as host genetics, comorbidities, and pre-existing immunity impact an individual’s viral kinetics and symptom development and thus also host infectiousness [11]. Here, we explore how pre-existing immunity of infected individuals may impact the transmission of a respiratory virus.

The role of pre-existing immunity in impacting virus spread has been increasingly studied over the last decade [12–15]. This immunity can stem from either natural infection or vaccination. Studies focused on respiratory viruses have shown that immunity can reduce an individual’s susceptibility to infection [16,17]. In the case of breakthrough infection, pre-existing immunity can reduce viral load, resulting in lower transmission [18]. Experimental transmission studies using animal models offer a controlled approach for quantifying the role of pre-existing immunity in impacting transmission outcomes [12,19] and dissecting the underlying mechanisms. These studies provide an opportunity to track the within-host viral dynamics of infected individuals that differ in their exposure histories and thus in their immunity. They also allow for quantitative assessment of how immunity impacts between-host transmission. Many different animal models have been used to study the transmission of respiratory viruses in experimental settings [12,20–24]. A subset of these studies have specifically examined the effect of pre-existing immunity in reducing viral transmission [12,19,25,26]. However, the majority of these studies have focused on antibody-mediated immunity rather than cellular immunity or have not been able to tease apart these two types of immunity in their impact on transmission.

Here, we specifically focus on quantifying the impact of pre-existing cellular immunity in donor individuals on their onward transmission potential of murine respirovirus using analyses of two different experimental transmission studies performed in the same laboratory. Murine respirovirus (Sendai virus, or SeV) is a respiratory virus of mice that, like influenza A viruses and coronaviruses, is both airborne- and contact-transmitted in mice. Both studies characterize onward SeV transmission from mice that are intranasally vaccinated with a T-cell vaccine (hereafter, “immune” mice) versus from placebo-vaccinated (hereafter “control”) mice to naive contact mice. Both of these studies benefit from *in vivo* imaging of viral infection in live mice to longitudinally quantify within-host infection burden. Previously, we showed that pre-existing CD8 T cell immunity can reduce both susceptibility to infection and onward transmission in this mouse model of SeV infection [27]. Here, by mapping the relationship between within-host infection burden and transmission probability, our analyses corroborate our previous findings that pre-existing cellular immunity reduces infection burden and therewith reduces the probability of onward transmission. More intriguingly, our analyses also indicate that cellular immunity results in lower infectiousness than expected once reduced infection burden has been accounted for. We interpret these findings in the context of a mathematical model that considers within-host viral and immune dynamics to shed light on the mechanisms by which pre-existing T-cell immunity may impact onward transmission potential.

## Results

### Pre-existing cellular immunity reduces onward transmission probability by reducing infection burden

**Fig 1:**
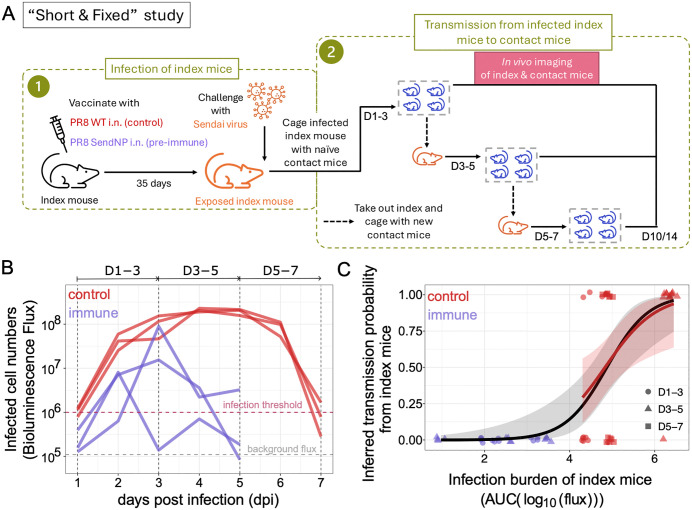
Infection dynamics and transmission outcomes from control and immune index mice in the “Short & Fixed” study. (A) Index mice were grouped into a control and an immune group. Mice in the control group were intranasally (i.n.) inoculated with a placebo vaccine (PR8 WT). Mice in the immune group were intranasally (i.n.) inoculated with a vaccine that elicited a cellular immune response to Sendai virus (PR8 SendNP). After 35 days, index mice from both the control group and the immune group were experimentally challenged with Sendai virus. Each index mouse was then sequentially placed in three cages, each of which had 4 naïve contact mice. The sequential placement of index mice occurred on days 1-3 (D1-3), days 3-5 (D3-5), and days 5-7 (D5-7). Infection dynamics in both index and contact mice were measured daily using bioluminescence flux. (B) Infection dynamics of control index mice (red) and immune index mice (purple). Infection levels are measured using bioluminescence flux (photons/sec) and plotted as a function of days post index mouse infection. Vertical dashed lines and the arrows on top of the figure panel specify the time windows when the index mice were co-housed with the naïve contact mice. (C) Transmission outcomes and estimated transmission probabilities as a function of index animal infection burden. Data points show transmission outcomes of individual contact mice (0 for no infection; 1 for infection), with their color reflecting whether the index mouse was from the control group (red) or from the immune group (purple) and their marker reflecting the time window of exposure. Infection status of contact mice are jittered across the infection burden of their corresponding index mouse to allow for individual data points to be seen. Logistic regression fit using all of data points is shown in black. The grey shaded regions show the 95% confidence intervals in the estimated transmission probabilities. Logistic regression using only the control data points is shown in red. On the log odds scale, the estimated parameters for the black curve are: intercept = -9.94 (SE: 2.93), slope = 2.04 (SE: 0.61); and the estimated parameters for the red curve are: intercept = -8.08 (SE: 3.33), slope = 1.67 (SE: 0.67).

Here, we first analyze data from a new transmission experiment and then perform more in-depth analysis of data from a previously published transmission experiment [27]. In the new experimental transmission study, infected index mice were placed in cages with naïve contact mice for a fixed time window of two days, with exposure to infected index mice occurring during different time windows following donor infection. Index mice comprised two different groups: an immune group that had been vaccinated with a Sendai Virus (SeV) T-cell vaccine and a control group that had been given a placebo vaccine that did not elicit a T-cell response to SeV. The experimental design is summarized in the *Materials & Methods* section and is schematically shown in Fig 1A. We hereafter refer to this study as the “Short & Fixed” study because contact mice were caged with index mice for short, fixed time periods of two days.

Fig 1B shows infection dynamics of the control and the immune index mice using *in vivo* measurements of bioluminescence flux, which can be interpreted as a measure of total infected cell numbers in an infected mouse. In the control mice, flux increased until 3–5 days post infection and then declined after day 5. By day 7, flux returned to levels that indicated resolution of infection. When the control index mice were caged with naïve contact mice during the 1–3 days-post infection time window (D1-3), their transmission efficiencies were 1/4, 3/4, and 1/4 in the three replicates performed (S1A Fig). When these same control index mice were caged with naïve contact mice during the D3-5 window, transmission efficiencies were 4/4, 4/4, and 3/4 (S1A Fig). Finally, during the D5-7 window, transmission efficiencies were 2/4, 1/4, and 3/4 in the three replicates (S1A Fig). Because infection levels are higher during the D3-5 window than in either the D1-3 window or the D5-7 window (Fig 1B), these results suggest that infection levels in a donor are positively related to transmission probability. To assess this possibility more quantitatively, we first calculated the infection burden for each of the control index mice for each of the three-time windows (see Materials & Methods). Infection burden of an index animal was calculated by taking the area under the curve (AUC) of the log of their infected cells dynamics over their time window of contact with a specific contact animal. We then plotted each contact’s infection outcome (0 for no infection; 1 for infection) against the infection burden of its corresponding index animal over the time period of its exposure (Fig 1C). A logistic regression model fit to these data indicates that transmission probability from an index animal is positively associated with its infection burden (Fig 1C) (p-value = 0*.*013).

We repeated this analysis for the three immune index mice. In these mice, flux again increased following infection, but peak flux occurred earlier (2–3 days post infection) and was generally lower than in the control index mice. Infection in immune animals also resolved earlier (by day 5) than in the control index mice. None of the three immune index animals transmitted SeV infection to any of the contact animals in either the D1-3 window or the D3-5 window (S1B Fig). A D5-7 window was not evaluated, given resolution of infection of the immune index animals by day 5. We calculated infection burdens for the immune index animals during the D1-3 windows and the D3-5 windows, and plotted the contacts’ infection outcomes (all 0) against the infection burdens of their corresponding index over the exposure time period (Fig 1C). Given the lack of transmission, we could not fit a logistic regression to these data alone. However, we could combine the transmission outcomes from the control index mice and the immune index mice and fit a logistic regression to this combined dataset. Doing so, we again found a significant positive association of transmission probability with total infection burden (Fig 1C) (p-value = 0*.*0008).

It is apparent from Fig 1C that pre-existing T-cell immunity reduces infection burden, which in turn results in a reduction of transmission probability. As such, the lower transmission efficiencies from immune index mice relative to those from control index mice appears to be, at least in part, driven by a reduced infection burden in the immune mice.

### Pre-existing cellular immunity reduces infectiousness in addition to infection burden

**Fig 2:**
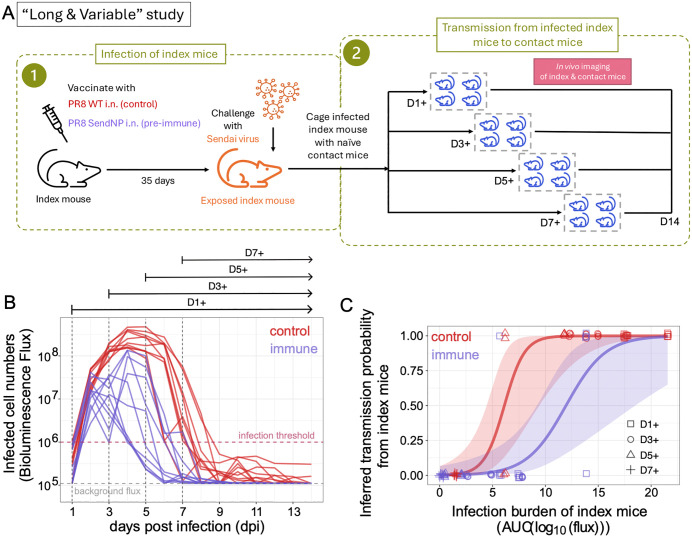
Infection dynamics and transmission outcomes from control and immune index mice in the “Long & Variable” study. (A) Index mice were grouped into a control and an immune group. Mice in the control group were intranasally (i.n.) inoculated with a placebo vaccine (PR8 WT). Mice in the immune group were intranasally (i.n.) inoculated with a vaccine that elicited a cellular immune response to Sendai virus (PR8 SendNP). After 35 days, index mice from both the control group and the immune group were experimentally challenged with Sendai virus. Each index mouse was then placed in a cage with 4 naïve contact mice starting at either at 1 (D1+), 3 (D3+), 5 (D5+), or 7 (D7+) days post infection (dpi). Infection dynamics in both index and contact mice were measured daily using bioluminescence flux. (B) Infection dynamics of control and immune index mice. Infection levels were measured using bioluminescence flux and plotted as a function of days post infection. Vertical dashed lines and the arrows on top of the figure panel specify the time windows when the index mice were co-housed with the naïve contact mice. (C) Transmission outcomes and estimated transmission probabilities as a function of index animal infection burden. Data points show transmission outcomes of individual contact mice (0 for no infection; 1 for infection), with their color reflecting whether their corresponding index mouse was from the control group (red) or from the immune group (purple) and their marker reflecting the time window of exposure. Infection status points of contact mice are jittered across the infection burden of their corresponding index mouse for visibility of all data points. A logistic regression model was fit to all the data points with immune status as a covariate to estimate the functional relationship between transmission probability and infection burden. On the log odds scale, the estimated parameters for the red curve are: intercept = -6.4 (SE: 1.9), slope = 1.04 (SE: 0.34). The estimated parameters for the purple curve are: intercept = -6.4 (SE: 1.9), slope = 0.53 (SE: 0.18). Both relationships are statistically significant: p-value = 0*.*002 for the control group and p-value = 0*.*003 for the immune group.

Because the “Short & Fixed” study resulted in no transmission from the immune index mice, we next considered a second study where the duration of exposure to both immune index mice and control index mice was extended, thereby increasing probabilities of onward transmission. Previously reported in [27], this second study considered three groups of index mice: a placebo-vaccinated control group (as in the “Short & Fixed” study) and two different immune groups. One immune group was vaccinated intranasally with the same T-cell SeV vaccine that was used in the “Short & Fixed” study. The second immune group was vaccinated intraparetonially with this vaccine. The experimental design of this study is further summarized in the *Materials & Methods* section and schematically shown in Fig 2A. Here, to parallel the index groups in the “Short & Fixed” study, we limited our analyses of this transmission study to the control group and the intranasally vaccinated immune group.

In this second study, infected index mice were transferred into cages with naïve contact mice either at 1-day post-infection (dpi) (D1+ group), at 3 dpi (D3+ group), at 5 dpi (D5+ group), or at 7 dpi (D7+ group). The index mice were then kept in their cages for the remainder of the experiment (up to 14 dpi). We hereafter refer to this study as the “Long & Variable” study because contact mice were caged with index mice for variable time periods spanning 7–13 days (longer than the 2 days in the “Short & Fixed” study). Fig 2B shows infection dynamics of the control and the immune index mice again using measurements of bioluminescence flux. As was observed in Fig 1B, flux in the control index mice increased until 4–5 days post infection and then started declining after day 5. Flux returned to levels that indicated resolution of infection by day 7–9. SeV transmission from the control index mice to naïve contact mice occurred during the D1+ , D3+ , and D5+ exposure windows with high transmission efficiency (S2A Fig). No onward transmission occurred during the D7+ exposure window (S2A Fig).

Consistent with the infection dynamics in the “Short & Fixed” study, flux in the immune index mice peaked earlier (2–4 days post infection) and at levels that were lower than in the control group (Fig 2B). Infection in immune animals again resolved earlier (by day 5–7). S2B Fig shows the infection dynamics of the contact mice alongside the infection dynamics of their corresponding immune index mice. Some transmission from immune index mice to contact mice occurred in the D1+ exposure window, but no transmission in D3+ and D5+ exposure windows was observed.

To quantify the relationship between infection burdens in the index mice and their onward transmission probabilities for this second transmission study, we first calculated the infection burden of the index mice during their respective transmission windows. We then plotted each contact’s infection outcome against the infection burden of its corresponding index animal for both control and immune groups (Fig 2C). Logistic regression models fit to these data again indicate that transmission probabilities are positively associated with infection burden. However, the extent of this association is different between the control and the immune groups (Fig 2C). Specifically, at intermediate index mouse infection burdens, it appears that the probability of transmission is lower from immune index mice than control index mice for the same infection burden.

**Fig 3:**
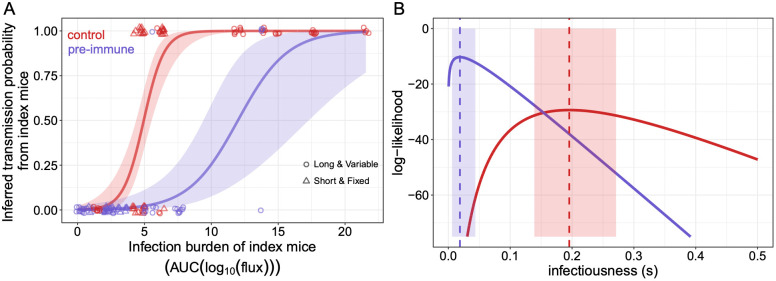
Transmission outcomes from and infectiousness of control and immune index mice when combining data from the “Short & Fixed” and “Long & Variable” studies. (A) Transmission outcomes and estimated transmission probabilities as a function of index animal infection burden. Data points show transmission outcomes of individual contact mice (0 for no infection; 1 for infection), with their color reflects whether the index mouse was from the control group (red) or from the immune group (purple) and their marker reflects the study from which the data points are derived. Infection status points of contact mice are jittered across the infection burden of their corresponding index mouse for visibility of all data points. A logistic regression model is fit to all the data points with index immune status as a covariate. On the log odds scale, the estimated parameters for the red curve are: intercept = -6.46 (SE: 1.4), slope = 1.31 (SE: 0.28). The estimated parameters for the purple curve are: intercept = -6.46 (SE: 1.4), slope = 0.54 (SE: 0.14). Both relationships are statistically significant: p-value = $3\times {\text{1}\text{0}}^{-\text{6}}$ for the control group and p-value = $2\times {\text{1}\text{0}}^{-\text{4}}$ for the immune group. (B) Estimates of infectiousness 𝐬 of the control index animals (red) and the immune index animals (purple). Red and purple dashed vertical lines show maximum likelihood estimates of the parameter 𝐬 for the control group (${𝐬}_{control}$) and the immune mice (${𝐬}_{immune}$), respectively. Maximum likelihood estimates are given by the values of  𝐬 that correspond to the peak values of the log-likelihood curves shown. 95% confidence intervals are shown as shaded regions surrounding the maximum likelihood estimates of  𝐬.

In a final analysis, we combined the available data from the “Short & Fixed” and the “Long & Variable” studies to ascertain the robustness of our results and further quantify the extent to which pre-immunity might reduce donor infectiousness. We again fit a logistic regression to infer the relationship between donor infection burden and transmission probability, this time with the combined data points (Fig 3A). Consistent with our previous results shown in Fig 2C, we find that the transmission probability from immune index animals is estimated to be lower than that of control index animals when controlling for infection burden. Additional analysis presented in S3 Fig and S1 Table, where we fit a logistic regression model in an immune-invariant framework, further supports the conclusions from Fig 3A. We note that our results depend on accurate assessment of whether transmission occurred or not. In both transmission studies, contact mice were assessed for infection for a substantial amount of time (10–14 dpi). Infection of contact mice is unlikely after this period because both control and immune index mice cleared infection by 8 dpi. We thus feel confident in our assessment of transmission occurrence.

These results indicate that infection burden is unlikely to be the sole predictor of transmission probability. Indeed, previous analyses of several experimental transmission studies have posited that infection stage, the onset of clinical manifestations, the virus’s ability to successfully establish a new infection, and the onset of the immune response might be additional factors that impact transmission probabilities [28–31]. When we divided the index infection burden into 4 bins ranging from 0-5, 5–10, 10–16, and 16–21, and calculated the fraction of contact mice infected in each bin, we see that both 0–5 and 5–10 infection burden bins show significant differences between the control and the immune groups (S4 Fig). Additionally, we saw a trending but non-significant increase in the time of transmission after an index mouse was transferred in a cage for transmission between the control and immune groups (mean transmission time from the control group = 3.9 days vs the mean transmission time from the immune group = 4.75 days, p-value = 0.073). This observation demonstrates that immune donor mice who end up transmitting virus to their corresponding contact mice tend to transmit later than the control donors, and indicates differences in infectiousness between these groups of mice. We therefore further quantitatively explored the extent to which pre-existing TRMs reduce host infectiousness using a time-varying force-of-infection (FOI) inference approach (see *Materials & Methods*). Here we considered a simple time-varying FOI approach that uses longitudinal measurements of infection load in an index animal and infection outcome of its corresponding contact animals to statistically estimate a parameter s that quantifies index infectiousness. Application of this approach to the data from the two transmission studies indicates that the infectiousness of an immune index mouse is lower than the infectiousness of a control index mouse (Fig 3B) ($scontrol=\text{0}.\text{1}\text{9}(\text{9}\text{5}\%CI:\text{0}.\text{1}\text{3}-\text{0}.\text{2}\text{6})\&s_{immune}=\text{0}.\text{0}\text{2}(\text{9}\text{5}\%CI:\text{0}.\text{0}\text{0}\text{0}\text{4}-\text{0}.\text{0}\text{4}))$.

In summary, an immune index mouse is expected to have a lower probability of transmission to a contact mouse than would a control index mouse that has the same measured infection burden. Moreover, conditional on successful transmission, transmission from an immune index mouse would be expected to occur later than from a control index mouse that has the same infection dynamics. Next, we use experimental observations along with mathematical modeling to explore possible reasons of the inferred differences in infectiousness.

### IFN-γ appears partly responsible for the inferred differences in infectiousness between control and immune groups

One possible reason for lower inferred infectiousness of the immune group could be related to the production of functional virus that can transmit from index mice to successfully infect contact mice. Production of virus from infected cells might be different between control and immune groups due to differences in the onset of host immune responses or the presence of pre-existing resident memory T cells. To evaluate this possibility, we therefore sought to correlate functional viral load, measured by plaque assay, to infected cell numbers, measured by bioluminescence flux, in the control and the immune groups. We did not find any evidence that the flux-PFU relationship was different when memory T cells were present versus when they were absent (S5 Fig). Pre-existing tissue resident memory T cells thus reduces virus replication and facilitates early clearance of the virus, which results in reduced infection burden of the immune host. This reduced infection burden is reflected in both flux (i.e., infected cell numbers) and PFU (i.e., functional viral load) measurements. After accounting for this reduced infection burden, intriguingly, we still infer an additional reduction in infectiousness from the immune group.

**Fig 4:**
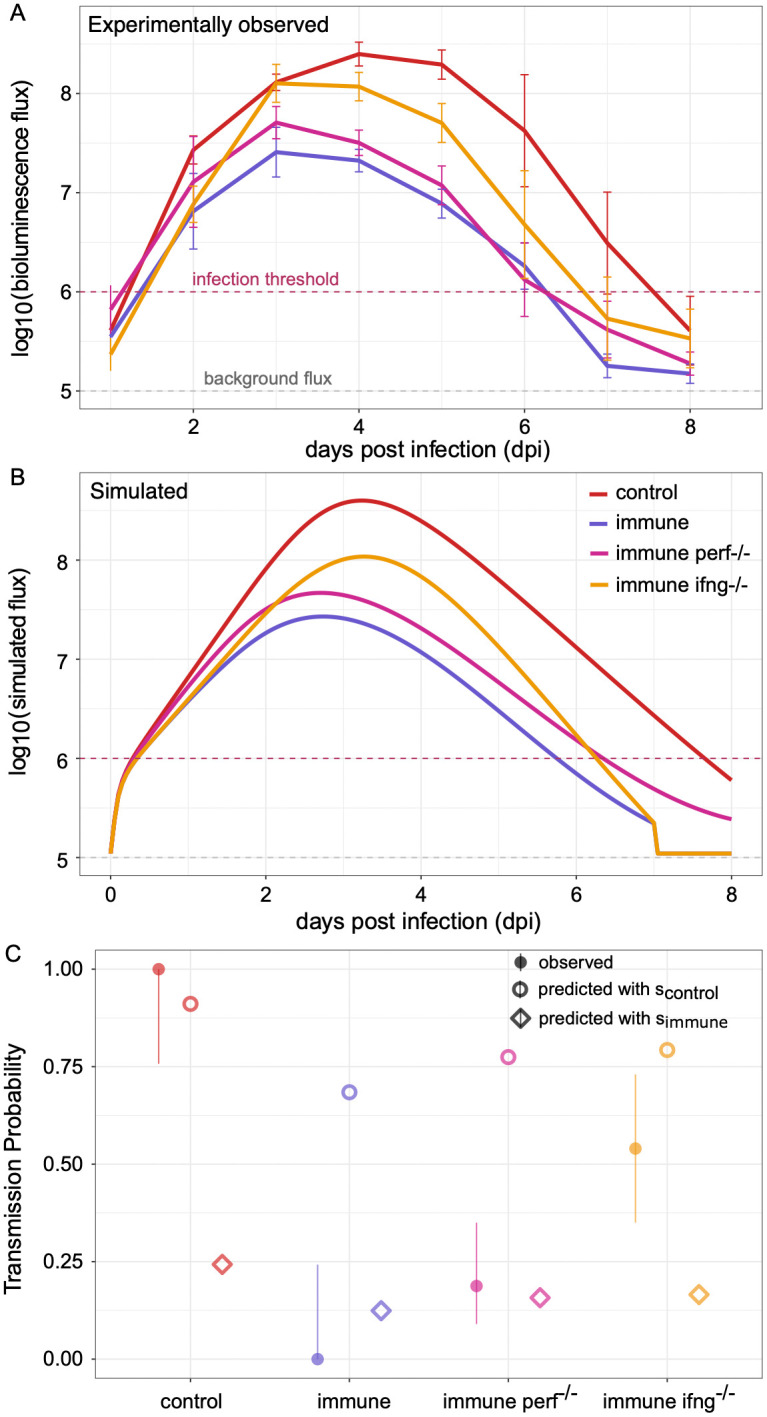
Within-host infection dynamics and transmission probability estimates under different experimental conditions. (A) Experimentally observed infection dynamics under different conditions are plotted in different colors. Color key is provided in panel B. (B) Simulated infection dynamics under these different conditions generated by changing parameters in our within-host model. (C) Observed (solid circles with error bars) and model-predicted (open markers) transmission probabilities for the D3+ group from the “Long & Variable” transmission study under different experimental conditions. $𝐩𝐞𝐫f^{-/-}$ was simulated by reducing the model parameter $\delta_{M}$. IFN-$\gamma^{-/-}$ was simulated by setting the T cell IFN-γ production rate to 0 in the within-host model. Transmission probabilities were estimated for the different immune groups using both ${𝐬}_{control}$ (open circles) and ${𝐬}_{immune}$ (open diamonds) to examine what causes reduced infectiousness in the immune group.

A second possible reason for the inferred differences in infectiousness could be the rapid production of IFN-γ by resident memory T cells following infection. Presence of CD8\ TRM cells in the immune group might render functional virus to be less infectious, for example, by secreting and transmitting IFN-γ when transmitting the virus. Here we quantitatively explore this possibility using mathematical modeling. We first consider a within-host mechanistic model of acute infection and then predict between-host transmission probabilities with the modeled infection dynamics. The mathematical description of the within-host model is provided in *Materials & Methods*. Briefly, uninfected target epithelial cells become infected with Sendai virus following exposure to free infectious virus. With a time-delay of ~1-day, infected cells release type-I interferon (IFN). Recognition of infected cells further stimulates existing tissue resident memory CD8 T cells (TRM). In turn, stimulated TRM cells produce effector molecules such as perforin (perf) and granzymes that directly kill infected cells. With a time-delay on the order of hours, interactions between infected cells and TRM cells (when present) result in IFN-γ production from stimulated T cells [32]. The overall IFN response in turn blocks virus production from infected cells. This model captures the essential mechanistic details following an acute viral infection, both in the absence and in the presence of existing TRMs. The model also reproduces key features of infected cell dynamics that we observe using bioluminescence flux in both the control and immune index mice (Fig 4A and 4B). Parameterized for immune index mice, this model predicts peak infected cell numbers that are about one order of magnitude smaller than the peak in control index mice. Model simulations further predict that the peak in immune mice occurs approximately a day earlier than that of control mice and that infected cell clearance also occurs earlier in immune mice. S6 Fig shows the dynamics of the other model variables. Furthermore, by combining our simulated flux dynamics with corresponding infectiousness profiles (estimated in Fig 3), we could reproduce observed patterns of onward transmission probabilities in both the “Short & Fixed” and “Long & Variable” studies (S7 Fig). For instance, in Fig 4C, we show that our transmission probability predictions for the D3+ group from the “Long & Variable” study reproduce observed transmission probabilities: with control mice, we predict close to 100% transmission probability, whereas with the immune mice, largely because $s_{immune}<s_{control}$, we predict only ~ 12% transmission probability. Overall, these results support our data analyses in Fig 3 and suggest that pre-existing TRMs reduced transmission probability to an extent that cannot be solely explained by reductions in infection burden.

We then used our model to quantitatively explore different mechanisms of protection conferred by tissue resident memory T cells in limiting transmission. Using the within-host model, we first simulated a case where perforin was knocked-out from immune mice (immune$perf^{-/-}$). Since perforin is not the only cytotoxic effector molecule by which CD8 T cells exhibit their cytolytic functions, we implemented $perf^{-/-}$ by reducing the T-cell killing rate ($\delta_{M}$) 3-fold in the model equations. Simulations of this model yields infected cell number dynamics that are similar to those simulated for the no knock-out immune group (Fig 4B), with only slightly higher infected cell numbers throughout the course of simulated infection. These model simulations tightly recover observed infection dynamics under $perf^{-/-}$ experimental conditions shown in Fig 4A. Under this knock-out scenario, using the estimated $s_{immune}$ from Fig 3B, we see only a slight increase in the predicted transmission probability from the immune group compared to what is observed under the no knock-out condition (purple vs magenta diamonds). This result is in line with transmission probabilities observed in the experiments by Uddbäck and colleagues [27], where they tracked transmission from $perf^{-/-}$ immune mice for the D3+ group in the “Long & Variable” transmission study (purple vs magenta solid circles with error bars). As such, the lower infectiousness of immune mice cannot be parsimoniously explained by the impact that tissue resident memory T cells have via perforin.

We next simulated a case where IFN-γ was knocked-out from the immune mice (immune IFN-$\gamma^{-/-}$). Since we assumed that IFN-γ is produced only by TRM cells when present, this knock-out was simulated by setting the T-cell IFN-γ production rate ($y_{M}$) to 0 in the model equations (see *Methods*). With IFN-$\gamma^{-/-}$, we see a more substantive increase in simulated infected cell numbers compared to the $perf^{-/-}$ simulations (Fig 4B). We further see a slightly delayed peak relative to simulations parameterized for the no knock-out immune group (Fig 4B). Our model’s predictions of higher infected cell numbers and a slightly delayed peak are reproduced under IFN-$\gamma^{-/-}$ knock-out experimental conditions (Fig 4A). We then again estimated transmission probabilities under this knock-out scenario by combining model simulations with infectiousness estimates from Fig 3B. Using the estimated $s_{immune}$ from Fig 3B, we predicted only a slightly increased transmission probability under $perf^{-/-}$ conditions in the immune group compared to no knock-out conditions in the immune group. This prediction, however, does not match the findings by Uddbäck and colleagues that reported a significantly increased transmission probability from the D3+ group with IFN-γ knock-out (Fig 4C). The discrepancy between the model and the data in the immune IFN-$\gamma^{-/-}$case suggests that IFN-γ has a larger effect than just reducing the infection load. In the absence of IFN-γ, infectiousness is increased in this group. By using the estimated $s_{control}$ from Fig 3B for the immune IFN-$\gamma^{-/-}$group, our prediction of transmission probability is only slightly higher than the observed transmission probability (open and solid golden yellow circles with error bars), suggesting that IFN-γ could be largely responsible for the observed differences in infectiousness between the control and immune groups.

## Discussion

Transmission of pathogens from infected individuals to uninfected hosts causes spread of infectious diseases in populations of individuals. In this study, we explored the role of immunity in preventing or limiting transmission of respiratory viruses. We used a combination of statistical data analyses and mathematical modeling to dissect the role of tissue-resident memory T cells (TRM) in limiting transmission of infectious virus between hosts. Specifically, we analyzed experimental data of Sendai virus (SeV) transmission in a mouse model in the presence and absence of TRMs.

Our earlier analysis of this transmission system suggested that with pre-existing TRMs, SeV transmission is curtailed earlier than without any T cell immunity [27]. There can be two ways in which TRMs can do this: by reducing the total infection burden that is required to transmit the virus, and by reducing the propensity to transmit infectious virus per unit infection burden for a given contact duration. Here we disentangled  these two possibilities by specifically linking within-host infection dynamics to between-host transmission. While it was reported that the presence of TRMs reduces the total infection burden to transmit, we report for the first time that TRMs may also reduce the propensity to transmit infectious virus per unit infection burden, which we call ‘infectiousness’.

Our different infectiousness estimates for the control and the immune groups were based on relating transmission probabilities to the log-transformation of the infection load, measured here as total bioluminescence flux. Earlier studies linking transmission rate to within host viral burden provide some empirical evidence in support of this functional form [33–35]. A recent study has explicitly tested different functional forms to link influenza transmission with infection load in a ferret model and found that a log and threshold FOI model performed similarly to recapitulate the observed data [36]. In S2 Table, we show that our general conclusion of reduced infectiousness in the immune group holds true if we linearly correlate transmission probability with infection load. It needs to be determined in future studies if other functional forms also provide similar results. Additionally, one can assume different functions to link transmission probability to the within-host FOI. In S3 Table, we provide likelihood values along with estimated parameters for such different functions. Regardless of the choice of functional form, fitting immune group specific transmission parameters was preferred in all the cases. The choice of functional form to link within-host infection dynamics to between-host transmission is an active research area and more mechanistic details about early infection processes will be required to derive an accurate mathematical description [31,37].

Using mathematical modeling, we further explored possible within-host immunological mechanisms that could be responsible for the apparent reduced infectiousness of immune mice. Earlier studies modeled acute infection dynamics, mainly to understand which processes are responsible for clearing infections [38,39]. Other studies have used models to estimate infection-related parameters that are difficult to quantify experimentally [40]. The model originally developed in Baccam et al. [40] was later extended to consider immune responses and additional complexities not captured by the initial model [41–43]. A recent study has specifically modeled the role of CD8 T cells in clearing influenza virus infection in mice and linked within-host viral dynamics to lung injury and disease severity [44]. Another study modeled SeV infection dynamics in mice that also reported bioluminescence flux and PFU titers to reflect infected cell and virus dynamics [45]. That study modeled the death rate of infected cells as a density-dependent term to reflect a biphasic decline of infection. Here we used a model structure similar to that reported in Baccam et al. [40] (see *Methods* for detailed model description and refer to S6D Fig for a visual representation of the model). Our rational in choosing this simple model is because (i) we do not see any biphasic infection clearance as observed in [45]; (ii) we wanted to model the effect mediated by respiratory tract resident TRMs which act mainly by producing IFN-γ [27,32]; (iii) in the absence of dynamical data on specific immune responses, we wanted to adopt the most parsimonious approach to capture the essential features of the within-host infection dynamics. The model was parameterized to reflect the experimentally observed infection dynamics. We would like to acknowledge that the assumptions in this mathematical model are broad, and biological systems are inherently variable. An important limitation to highlight here is that these simple dynamical models use mean field approximation for the biological processes governing infection and immunity, and ignore any heterogeneity (e.g., spatial) during the infection process. Spatial heterogeneity may need to be accounted for when modeling infection dynamics in low/high initial Sendai virus dose [46]. This type of in-depth modeling is not within the scope of this current study. Additionally, transmission from index animals may depend on low versus high inoculum dose to start the infection process due to heterogeneity. Hence, future studies should quantify any such heterogeneity in the infection process which might be informative during vaccine regimen development.

The purpose of the simulation modeling was to mechanistically capture patterns seen in the data collected at different scales, that is, both within hosts and transmission between hosts. Building on previous studies that have aimed to quantitatively link within-host infection dynamics to transmission outcomes [29–31,47,48], we here linked within-host infection dynamics to transmission probabilities for SeV in mice using a force of infection (FOI) framework. While our models could broadly recapitulate experimentally observed transmission dynamics (S7 Fig), we would like to note that our modeling framework was not developed to match the actual numbers of different quantities that were measured experimentally. Transmission probability estimates in the experiments are not exactly the same as we predicted and could be simply due to nonrandom contact patterns in a cage and other transmission-related heterogeneity [49,50]. Further, when predicting transmission probability, we used only a single set of within-host model parameters along with single values for infectiousness (either $s_{control}$ or $s_{immune}$). Almost certainly there will be variability in these parameters, which will impact the predicted mean transmission probability. Nevertheless, the data analysis and the model presented in this study serve two purposes. First, by reproducing the observed kinetic patterns of within-host infection burden and between host transmission outcomes, the model provides a quantitative basis for the observed data at multiscale level. Second, the model is consistent with the observation that IFN-γ may be partially responsible for the reduced ‘infectiousness’ supporting the findings of the knock-out experiments.

Acute infections are hypothesized to be controlled and eventually cleared primarily by innate immunity, such as interferon, NK cells, and recruitment of other inflammatory cytokines, in the absence of an adaptive immune memory [51,52]. Adaptive immune memory, i.e., antibodies or memory CD8 T cells, may further facilitate early clearance of infection and limit transmission [27,53–56]. Earlier studies have looked into the role of pre-existing antibody responses in reducing SeV reinfection in mice, following their exposure to SeV by different transmission modes [57,58]. In our system, no pre-existing SeV specific memory B cells were present. This allowed us to specifically look at the role of the memory CD8 T cell response. Previously a study by Price and colleagues investigated the role of CD8 T cells in limiting transmission of influenza viruses [59]. They found that a vaccine, inducing a T-cell response, could limit virus replication in the nasal cavity compared to unvaccinated animals, whereas virus dynamics in the lung was similar between these groups. Importantly, they observed significantly reduced transmission from the T-cell vaccine group. T cells play crucial roles in clearance of virus infections by directly killing infected cells and by producing antiviral cytokines. Moreover, an interferon response is stimulated by virus-infected cells, and pre-existing T cells can further bolster this effect. Zhou et al. [60] tested the role of IFN-γ in blocking infection in the presence of SeV specific CD8 T cells generated by Ad-SenNP vaccination. They found that CD8 T cells mediate early resistance to viral challenge primarily through release of IFN−γ. Our findings of reduced infectiousness (and essentially no transmission at early time points following challenge infection) along with reduced infection burden in the immune group could also be due to an early IFN−γ response that might reduce the infectious viral load even if virus is detected using a plaque assay. Viral infectivity in plaque assays is highly dependent on the target cells used for infection and functional virus titer inferred by *in vitro* measurements may not reflect the titer on cells *in vivo*. Further, it is poorly understood how functional virus detected by plaque assay relates to infectiousness of an individual leading to effective between host transmission [11,28]. The exact biological mechanisms of how IFN−γ modifies immune donor infectiousness is unclear, and future studies should address this question in more detail. A T cell-specific IFN$-\gamma^{-/-}$ or IFN$-{\gamma R}^{-/-}$ experiment will be needed to directly test the role of IFN−γ produced by the TRM cells in limiting transmission. Additionally, quantitative measurement of infectious virus, capable of establishing infection following transmission, in knock-out vs no knock-out scenarios need to be performed.

T cells provide important antiviral defense and also target evolutionary conserved epitopes on respiratory viral genomes. This makes them an excellent candidate for incorporating into the design of universal vaccines against rapidly evolving respiratory viruses. The role of T cell mediated protection against respiratory viruses is understudied compared to the role of antibodies. Our earlier study along with this study focused on understanding the role of T cells in limiting transmission and reducing susceptibility to infection. However, we have used mouse and murine para influenza virus as our model system. Whether similar findings can be obtained in other animal models and other respiratory viruses needs to be determined. Future studies investigating the role of T cells in other animal models that can closely reflect respiratory virus transmission in humans are therefore needed to assess whether and to what extent T cell immunity can protect against infection and onward transmission potential.

## Materials and methods

### Experimental transmission study details

The “Short & Fixed” transmission study and the previously published “Long & Variable” transmission study were both conducted in the Kohlmeier research laboratory at Emory University. Details on the “Long & Variable” transmission study are already provided in [27]. The “Short & Fixed” transmission study also used six- to eight-week-old female C57BL/6 mice obtained from Jackson Laboratory and housed them under the same pathogen free conditions at Emory University. Both transmission study experiments were completed in accordance with the Institutional Animal Care and Use Committee guidelines of Emory University, PROTO201700581. Age matched mice were randomly assigned to experimental groups for both experiments.

For intranasal priming, 30 plaque-forming units (PFU) Influenza A/Puerto Rico/8/34 (PR8-WT), 50 PFU Influenza A/Puerto Rico/8/34 expressing Sendai nucleoprotein FAPGNYPAL epitope (PR8-SenNP) were administered in a 30 μL volume under isoflurane anaesthesia (Patterson Veterinary). Encoding luciferase SeV (Sendai-Luc) was generated and grown as previously described [27]. For direct Sendai-Luc infection, 1500 PFU in 30 μL was administered intranasally under isoflurane anaesthesia.

*In vivo* imaging was done using an In Vivo Imaging System (IVIS) Lumina LT Series III (Perkin Elmer) with an XFOV-24 lens as previously described [27]. Bioluminescent signal was quantified by manually drawing regions of interest around the respiratory tract using known anatomical markers. Bioluminescence in this system quantifies the number of infected cells, rather than extracellular virus, as it relies on reporter gene expression and is only detectable when the viral genome is translated.

For longitudinal detection of viral titers from the nasal cavity of infected mice, virus was collected by dipping the nose of each mouse into 1% BSA in PBS 20 times in a 12-well plate under isoflurane anaesthesia as described previously [27]. Samples were collected in duplicates. Plaques were counted using the following formula: PFU/ml = (average number of plaques) × (dilution fold).

**Transmission study design:** We designed a transmission experiment setup where SeV transmission to naïve contact mice from an immune or a naïve index mouse is tracked for a short and fixed contact duration distributed throughout its entire infection period. Through-out this paper, we refer to this experiment as the “Short & Fixed” duration transmission experiment (experimental design shown in Fig 1A). As depicted in the schematic, we first vaccinated mice intranasally with PR8 WT or with PR8 SendNP recombinant vaccine. Vaccination with PR8 WT generated no SeV specific T cell immunity, whereas the PR8 SendNP i.n. vaccination generated Sendai NP specific resident memory CD8 T (TRM) cells [27]. 35 days following vaccination, mice were artificially infected with SeV. Each infected index mouse was sequentially co-housed with groups of four naive contact mice for consecutive 48-hour intervals. Specifically, beginning at day 1 post infection (dpi), index mice were placed in a clean cage with a first group of contact mice for 2 days (1–3 dpi). Following the 48-hour exposure period, index mice were then transferred to a new clean cage with a fresh group of naive contact mice for an additional 2 days (3–5 dpi), and then transferred to the third and final new cage of fresh contact mice between 5–7 dpi. Thus, transmission from index mice was tracked within 1–3 days post infection (dpi) (D1-3 group), and then within 3–5 dpi (D3-5 group), and finally within 5–7 dpi (D5-7 group). Since the immune index mice cleared the infection (defined by bioluminescence flux level below the infection threshold) at day 5, transmission was not tracked afterwards.

The second transmission study, referred to as “Long & Variable” duration transmission is schematically represented in Fig 2A. The detailed description of this transmission experiment is provided in our previous work by Uddbäck and Michalets et al. [27]. For the inoculum dose of SeV that we used to infect index animals, weight loss of infected animals was minimal (around 8% at 8 dpi) and returned to baseline weight within 12 days, consistent with very little morbidity/disease upon infection. Following this observation, one can expect that there will be little (if any) difference in contact rates between infected and uninfected animals as infection induced morbidity is minimum. However, we currently do not know if contact rate or any other symptoms, e.g., sneezing or coughing, changed between immunized and placebo groups following infections.

### Data analysis and modelling of transmission experiments

#### Logistic regression, transmission probability, and infectiousness calculations.

We first calculated the area under the curve (AUC) of the bioluminescent signal, measured by the IVIS, of an index mouse from the time when the mouse was caged with contact mice to the time the mouse was kept in the cage to track transmission. During the contact period between an index mouse and its corresponding contact mice in each cage, we then analyzed which of the 4 contact mice got infected using a threshold of infection ${\text{log}}_{\text{1}\text{0}}(flux)=\text{6}$. The threshold of infection was estimated as the 95% CI of the background bioluminescent signal from two uninfected mice [27]. By tracking each contact mouse placed in a given cage with a given index mouse, we could then estimate the probability of transmission from that index mouse as a function of its AUC and immune status using a simple logistic regression model. Fit to the logistic regression was performed using the R function *glm()* with family as *binomial* and link as logit. More specifically, here, probability of transmission is calculated as a two-parameter logistic function with intercept a and slope b according to the formula


$$P_{trans}=\frac{e^{a+b\times infectionburden}}{\text{1}+e^{a+b\times infectionburden}}$$ (1)

where infection burden is the AUC of the ${\text{log}}_{\text{1}\text{0}}(flux)$ for the corresponding exposure duration. As shown in S1 Table, we have considered different forms of the logistic regression model to fit to the data presented in Fig 3A. We also considered infection burden to be AUC(flux) for an index animal’s transmission window and results are shown in S1 and S2 Tables.

For infectiousness calculations, assuming transmission from an index to a contact follows a poisson process,


$$P_{trans}=\text{1}-e^{-FOI(t)}$$ (2)

We consider a simple definition of the FOI(t) for a given transmission window t1 to t2 as $s\int_{t\text{1}}^{t\text{2}}{\text{log}}_{\text{10}}(infectionload)dt$, where *s* is infectiousness. This translates to taking the AUC of the log10 infected cell numbers (or flux) of an index mouse, i.e., the animal’s infection burden for it’s given transmission window. In this formulation of modeling transmission as a poisson process, s is equivalent to b in the logistic model described above in equation 1. The parameter s is then estimated using maximum likelihood [61] by calculating the probabilities of the contact animals having been infected when they are caged with an index mouse across a range of s values. For all these calculations, the background AUC which is determined by the background flux of 10^5^ was subtracted from the calculated AUCs to infer the effect of actual infection burden. S2 Table compares different transmission models fitted to the data presented in Fig 3A.

Any contact mouse, that showed detected infection by crossing the threshold of infection more than 3 days after their corresponding index mouse was removed or cleared infection, was considered uninfected by the index mouse in our analysis. Our results are, however, robust to this assumption and hold true even if these contact mice with delayed infection are considered to be infected by the index animals.

#### Within-host mathematical modeling.

Here we use a simple mathematical modelling framework analogus to the target cell limitation model, borrowed from the literature [40]. The model equations are as follows,


$$\frac{dT}{dt}=-\beta VT$$ (3)

$$\frac{dI}{dt}=\beta VT-\delta I-\delta_{M}IM$$ (4)

$$\frac{dV}{dt}=\frac{pI}{\text{1}+\zeta F}-cV$$ (5)

$$\frac{dF}{dt}=xI\left(t-\tau_{\text{1}}\right)+y_{M}I\left(t-\tau_{\text{2}}\right)-\alpha F$$ (6)

The target cell population 𝑚 is infected by the virus 𝑜 following a mass-action law with an infectivity of β. Upon infection of uninfected cells, infected cells can be cleared at a per capita rate of δ. Infected cells can also be killed by the TRM cells (M) at a second order rate constant $\delta_{M}$. Infected cells produce virus at a per capita rate of p. However, the production rate of virus from infected cells might be reduced in the presence of interferon, modelled here as F. The effect of interferon on the virus production rate is modelled using the term $\frac{pI}{\text{1}+\zeta F}$ where ζ is efficacy of interferon in blocking virus production from the infected cells. Note, here we do not model M as a dynamical equation since previous study by McMaster et al. [32] reported that upon infection the TRM cell population doesn’t divide and proliferate, rather act by secreting IFN-γ rapidly. Finally, we model the type-I interferon (IFN) response to accumulate following a time delay of $\tau_{\text{1}}$ proportional to the infected cell population at (${t-\tau }_{\text{1}}$), and IFN-γ to be produced by the TRM cells (M) following a time delay of $\tau_{\text{2}}$ when TRM cells are present. IFN-γ production is also proportional to the infected cell numbers at (${t-\tau }_{\text{2}}$), but depends on the TRM cell numbers (M). Interferon (both type-I and IFN-γ) is cleared at a per capita rate of α. In this model, we have lumped the effects of type-I and IFN-γ into a single equation of F. It might be the case that these two interferon subtypes play different roles and show different kinetics. But in the absence of more granular data on interferon dynamics, we preferred this approach.

The infection related parameters to simulate this model were partly informed by Pinky et al. [45]. Note that we do not use the exact same model structure or the same parameter values as used in Pinky et al. [45] since we do not see a biphasic clearance pattern of infection in the mouse strain that was used in our study. Different infection kinetics with differences in inoculum dose and mouse strain was reported earlier in Burke et al. [57]. Parameters related to IFN dynamics have been informed by work done by Handel and colleagues [62], since they modeled influenza infection in mice accounting for IFN dynamics. Specifically, we use the following parameter values: $\beta =\text{1}\times {\text{1}\text{0}}^{-\text{4}}{\left(virus\right)}^{-\text{1}}{\left(day\right)}^{-\text{1}}$, $p=\text{2}.\text{8}{\left(cell\right)}^{-\text{1}}{\left(day\right)}^{-\text{1}}$, $c=\text{4}{\left(day\right)}^{-\text{1}}$, $\tau_{\text{1}}=\text{1}.\text{5}day$,$\tau_{\text{2}}=\text{0}.\text{2}day$, $\delta =\text{1}.\text{7}{\left(day\right)}^{-\text{1}}$, $\delta_{M}=\text{0}.\text{0}\text{0}\text{3}{\left(day\right)}^{-\text{1}}{\left(cell\right)}^{-\text{1}}$, x=0.018, $y_{M}=\text{0}.\text{0}\text{0}\text{5}\times \frac{M}{m+M}$, $\alpha =\text{0}.\text{8}(da{y)}^{-\text{1}}$, ζ=2, m=10cells. The model was simulated with the following initial conditions: $T_{\text{0}}={\text{1}\text{0}}^{\text{5}}cells$, $V_{\text{0}}=\text{5}$, $I_{\text{0}}=\text{0}$, $F_{\text{0}}=\text{0}$, and M=300cells [27] if there is pre-existing TRM cells, otherwise M=0.

Following initiation of an infection, simulations were stopped when the infected cell numbers went below 2 cells reflecting stochastic extinction and/or elimination of a small number of infected cells by the pre-existing TRM cells. After simulating the model with the above-mentioned parameters, we calculated simulated flux using ${\text{log}}_{\text{1}\text{0}}(simulatedflux)={\text{log}}_{\text{1}\text{0}}(I)+{\text{log}}_{\text{1}\text{0}}\left(backgroundflux\right)$. We then calculated the transmission probabilities for any exposure window by integrating the simulated flux within that window following the FOI framework using equation 2.

## Supporting information

S1 Fig Paired infection dynamics of infected index mice and their corresponding contacts in the “Short & Fixed” study. (A) Paired infection dynamics of the control index mice and their contacts. (B) Paired infection dynamics of the immune index mice and their contacts. Each panel (representing a single cage) shows flux of index (solid orange and dashed black lines) and contact (blue lines) animals as a function of days post infection of the index animals. The red horizontal dashed line in each panel shows the bioluminescence flux threshold for calling infection. The grey horizontal dashed line in each panel shows the mean background level of bioluminescence flux. Solid orange lines in the index mice infection dynamics represent their exposure duration in each transmission window, i.e., D1-3, D3-5, and D5-7. Numbers of infected contact mice are indicated in each panel after discarding the ones who got infected more than 3 days following the removal of the index animal from a cage.
(DOCX)

S2 Fig Paired infection dynamics of infected index mice and their corresponding contacts in the “Long & Variable” study. (A) Paired infection dynamics of the control index mice and their contacts. (B) Paired infection dynamics of the immune index mice and their contacts. Each panel (representing a single cage) shows flux of index (solid orange and dashed black lines) and contact (blue lines) animals as a function of days post infection of the index animals. The red horizontal line in each panel shows the bioluminescence flux threshold for calling infection. The grey horizontal line in each panel shows the mean background level of bioluminescence flux. Solid orange lines in the index mice infection dynamics represent their exposure duration in each transmission window, i.e., D1+, D3+, D5+, and D7+. Numbers of infected contact mice are indicated in each panel after discarding the ones who got infected more than 3 days following infection clearance of the index animal in a cage.
(DOCX)

S3 Fig Comparison of logistic regression fits using two different methods. The black curve with its 95% CI as the grey shaded area was fitted with only one set of parameters ignoring donor immune status. For the black curve, in log-odds scale, estimated intercept: -3.7 (SE: 0.62), and estimated slope: 0.55 (SE: 0.1). AIC of this model fit is 105. A logistic regression fit using immune group specific slopes significantly improved the AIC to 66. The estimated parameters for this model is reported in the main text. Comparing the black curve with the corresponding-colored curves (red for the control and purple for the immune) suggests that the difference in transmission probabilities between the control and immune mice intensifies for an intermediate index infection burden (AUC between 5–10). We note here the lack of observed data points for infection burdens ranging from 7-11. However, these curves were inferred based on the entire range of observed infection burden, with non-overlapping CIs for most of this range.
(DOCX)

S4 Fig Fraction of contact mice infected as a function of index infection burden. We divide the AUC(log10(flux)) of the index animals or index infection burden into 4 bins ranging from 0-5, 5–10, 10–16, and 16–21, and calculate the fraction of contact mice infected in each bin. P-values indicate whether there is any significant difference in the fraction infected between immune groups for each bin.
(DOCX)

S5 FigRelationship between the number of infected cells (measured using bioluminescence flux) and the amount of infectious virus (measured using plaque assays).(A) We tested the relationship between the plaque forming units (a measure of functional virus) and the IVIS flux levels (a measure of infected cell numbers) from paired data where the nasal wash was collected and bioluminescence flux was measured from the same mouse at the same dpi. With pre-existing T cell memory, the bioluminescence flux levels are decreased, and we see an equivalent decrease in the measured levels of plaque forming units. We would like to note here that the PFU measurements are noisy and the adjusted $R^{\text{2}}$ value for an ordinary lease square fit (solid black line) to this data is 0.39. But log10(flux) is significantly associated with predicting log10(PFU/mL) as indicated by the p-values. We also performed a “Robust Regression” (dashed black line) and compared the estimated coefficients of this regression with that of ordinary least square. Visually, the robust linear model fit is not that different from the ordinary least square fit. Additionally, fitting 2 different lines separately for the control (indicated by the red line) and immune groups (indicated by the purple line) do not look different from the single black line. (B) The data used for this analysis was taken from Uddback et al. 2024. A group of vaccinated (PR8 SendNP i.n.) and unvaccinated (PR8 WT i.n.) mice were infected and daily measurements of flux using IVIS were performed. A separate group of vaccinated or unvaccinated mice were infected and daily measurements of PFU/mL were performed using the nasal wash plaque assay. Hence, here the measurements for flux and PFU/mL do not come from the same mouse. A linear relationship fitted between the mean ${log}_{\text{1}\text{0}}\left(flux\right)$and the mean ${log}_{\text{1}\text{0}}\left(PFU/mL\right)$ is indicated by the black line (adjusted $R^{\text{2}}=\text{0}.\text{8}\text{8}\text{4})$.(DOCX)

S6 Fig Simulations of within-host viral and immune dynamics. (A) Dynamics of target cells (blue), infected cells (red), viral load (magenta), and interferon (olive green) from the within-host model simulated with’control’ parameters. (B) Dynamics of target cells (blue), infected cells (red), viral load (magenta), and interferon (olive green) from the within-host model simulated with’immune’ parameters. (C) The relationship between simulated infected cell numbers and viral load from the control simulation (A) and immune simulation (B). In the immune group, we see lower infected cell and correspondingly lower viral load compared to the control group. However, the two groups show similar patterns in relating viral load to total infected cell numbers. (D) Diagram of the model showing interactions between different variables.
(DOCX)

S7 FigModel prediction of transmission experiment data.(A) Comparison between observed and predicted transmission probabilities for the “Short & Fixed” study and (B) “Long & Variable” study. Experimental data was not available for the D5-7 transmission window for the immune group. For the immune group, we used both $s_{control}$(in open circles) and $s_{immune}$(in open diamonds) to predict transmission probability with simulated immune infection dynamics. This allowed us to study the effect of TRMs on the infection burden alone, keeping infectiousness the same as the control group. As seen in the plots, for both transmission experiments, a reduction in infectiousness for the pre-immune group was necessary to predict the observed transmission probabilities.(DOCX)

S1 TableComparison of logistic regression fits to the combined dataset of “Short & Fixed” and “Long & Variable” transmission studies (data presented in Fig 3A).In all the below cases, we fit $P_{trans}=\frac{e^{a+b\times infectionburden}}{\text{1}+e^{a+b\times infectionburden}}$. We see that a logistic regression fit with infection burden to be calculated as AUC (log10(flux)) and parameter b being immune-group specific gives the best model fit to the data.(DOCX)

S2 Table Comparison of FOI forms in explaining the observed data in Fig 3A. Below we see that regardless of the form of FOI to model transmission probability, a model with immune-group specific parameters is always favored according to the maximum log-likelihood values.
(DOCX)

S3 Table Comparison of different transmission models fitted to the data presented in Fig 3A. Infection burden in all the below cases is calculated as the AUC(log10(flux)) for an index animal’s transmission window. we see that a logistic regression fit with immune group specific parameters performed equally well to the 2-parameter exponential model fit with immune group-specific parameters.
(DOCX)

S4 Table Raw data containing flux measurements for all the index and contact animals in both “short & fixed” and “long & variable” duration transmission studies. The data includes experimentally observed flux dynamics in knock-out groups, observed and predicted transmission probabilities, and paired and mean flux and PFU measurements.
(XLSX)
