# Supplementary material for: Quantifying the role of pre-existing tissue resident cellular immunity in limiting respiratory virus transmission
Source: PLoS Pathog. 2026 Apr 21;22(4):e1014082. doi: 10.1371/journal.ppat.1014082 (PMC13143178; doi:10.1371/journal.ppat.1014082)
Supplement: S1 Fig — (A) Paired infection dynamics of the control index mice and their contacts. (B) Paired infection dynamics of the immune index mice and their contacts. Each panel (representing a single cage) shows flux of index (solid orange and dashed black lines) and contact (blue lines) animals as a function of days post infection of the index animals. The red horizontal dashed line in each panel shows the bioluminescence flux threshold for calling infection. The grey horizontal dashed line in each panel shows the mean background level of bioluminescence flux. Solid orange lines in the index mice infection dynamics represent their exposure duration in each transmission window, i.e., D1-3, D3-5, and D5-7. Numbers of infected contact mice are indicated in each panel after discarding the ones who got infected more than 3 days following the removal of the index animal from a cage. (DOCX) [file ppat.1014082.s001.docx]

**S1 Fig**: **Paired infection dynamics of infected index mice and their corresponding contacts in the “Short & Fixed” study. (A)** Paired infection dynamics of the control index mice and their contacts. **(B)** Paired infection dynamics of the immune index mice and their contacts. Each panel (representing a single cage) shows flux of index (solid orange and dashed black lines) and contact (blue lines) animals as a function of days post infection of the index animals. The red horizontal dashed line in each panel shows the bioluminescence flux threshold for calling infection. The grey horizontal dashed line in each panel shows the mean background level of bioluminescence flux. Solid orange lines in the index mice infection dynamics represent their exposure duration in each transmission window, i.e., D1-3, D3-5, and D5-7. Numbers of infected contact mice are indicated in each panel after discarding the ones who got infected more than 3 days following the removal of the index animal from a cage.
