# Supplementary material for: Quantifying the role of pre-existing tissue resident cellular immunity in limiting respiratory virus transmission
Source: PLoS Pathog. 2026 Apr 21;22(4):e1014082. doi: 10.1371/journal.ppat.1014082 (PMC13143178; doi:10.1371/journal.ppat.1014082)
Supplement: S3 Fig — The black curve with its 95% CI as the grey shaded area was fitted with only one set of parameters ignoring donor immune status. For the black curve, in log-odds scale, estimated intercept: -3.7 (SE: 0.62), and estimated slope: 0.55 (SE: 0.1). AIC of this model fit is 105. A logistic regression fit using immune group specific slopes significantly improved the AIC to 66. The estimated parameters for this model is reported in the main text. Comparing the black curve with the corresponding-colored curves (red for the control and purple for the immune) suggests that the difference in transmission probabilities between the control and immune mice intensifies for an intermediate index infection burden (AUC between 5–10). We note here the lack of observed data points for infection burdens ranging from 7-11. However, these curves were inferred based on the entire range of observed infection burden, with non-overlapping CIs for most of this range. (DOCX) [file ppat.1014082.s003.docx]

**S3 Fig: Comparison of logistic regression fits using two different methods.** The black curve with its 95% CI as the grey shaded area was fitted with only one set of parameters ignoring donor immune status. For the black curve, in log-odds scale, estimated intercept: -3.7 (SE: 0.62), and estimated slope: 0.55 (SE: 0.1). AIC of this model fit is 105. A logistic regression fit using immune group specific slopes significantly improved the AIC to 66. The estimated parameters for this model is reported in the main text. Comparing the black curve with the corresponding-colored curves (red for the control and purple for the immune) suggests that the difference in transmission probabilities between the control and immune mice intensifies for an intermediate index infection burden (AUC between 5 to 10). We note here the lack of observed data points for infection burdens ranging from 7-11. However, these curves were inferred based on the entire range of observed infection burden, with non-overlapping CIs for most of this range.
