# Supplementary material for: Quantifying the role of pre-existing tissue resident cellular immunity in limiting respiratory virus transmission
Source: PLoS Pathog. 2026 Apr 21;22(4):e1014082. doi: 10.1371/journal.ppat.1014082 (PMC13143178; doi:10.1371/journal.ppat.1014082)
Supplement: S4 Fig — We divide the AUC(log10(flux)) of the index animals or index infection burden into 4 bins ranging from 0-5, 5–10, 10–16, and 16–21, and calculate the fraction of contact mice infected in each bin. P-values indicate whether there is any significant difference in the fraction infected between immune groups for each bin. (DOCX) [file ppat.1014082.s004.docx]

**S4 Fig: Fraction of contact mice infected as a function of index infection burden.** we divide the AUC(log10(flux)) of the index animals or index infection burden into 4 bins ranging from 0-5, 5-10, 10-16, and 16-21, and calculate the fraction of contact mice infected in each bin. P-values indicate whether there is any significant difference in the fraction infected between immune and control groups for each bin.
