# Supplementary material for: Quantifying the role of pre-existing tissue resident cellular immunity in limiting respiratory virus transmission
Source: PLoS Pathog. 2026 Apr 21;22(4):e1014082. doi: 10.1371/journal.ppat.1014082 (PMC13143178; doi:10.1371/journal.ppat.1014082)
Supplement: S5 Fig — (A) We tested the relationship between the plaque forming units (a measure of functional virus) and the IVIS flux levels (a measure of infected cell numbers) from paired data where the nasal wash was collected and bioluminescence flux was measured from the same mouse at the same dpi. With pre-existing T cell memory, the bioluminescence flux levels are decreased, and we see an equivalent decrease in the measured levels of plaque forming units. We would like to note here that the PFU measurements are noisy and the adjusted R2 value for an ordinary lease square fit (solid black line) to this data is 0.39. But log10(flux) is significantly associated with predicting log10(PFU/mL) as indicated by the p-values. We also performed a “Robust Regression” (dashed black line) and compared the estimated coefficients of this regression with that of ordinary least square. Visually, the robust linear model fit is not that different from the ordinary least square fit. Additionally, fitting 2 different lines separately for the control (indicated by the red line) and immune groups (indicated by the purple line) do not look different from the single black line. (B) The data used for this analysis was taken from Uddback et al. 2024. A group of vaccinated (PR8 SendNP i.n.) and unvaccinated (PR8 WT i.n.) mice were infected and daily measurements of flux using IVIS were performed. A separate group of vaccinated or unvaccinated mice were infected and daily measurements of PFU/mL were performed using the nasal wash plaque assay. Hence, here the measurements for flux and PFU/mL do not come from the same mouse. A linear relationship fitted between the mean log10(flux)and the mean log10(PFU/mL) is indicated by the black line (adjusted R2=0.884). (DOCX) [file ppat.1014082.s005.docx]

**S5 Fig**: **Relationship between the number of infected cells (measured using bioluminescence flux) and the amount of infectious virus (measured using plaque assays).** (**A)** We tested the relationship between the plaque forming units (a measure of functional virus) and the IVIS flux levels (a measure of infected cell numbers) from paired data where the nasal wash was collected and bioluminescence flux was measured from the same mouse at the same dpi. With pre-existing T cell memory, the bioluminescence flux levels are decreased, and we see an equivalent decrease in the measured levels of plaque forming units. We would like to note here that the PFU measurements are noisy and the adjusted $R^{2}$ value for an ordinary lease square fit (solid black line) to this data is 0.39. But log10(flux) is significantly associated with predicting log10(PFU/mL) as indicated by the p-values. We also performed a “Robust Regression” (dashed black line) and compared the estimated coefficients of this regression with that of ordinary least square. Visually, the robust linear model fit is not that different from the ordinary least square fit. Additionally, fitting 2 different lines separately for the control (indicated by the red line) and immune groups (indicated by the purple line) do not look different from the single black line. **(B)** The data used for this analysis was taken from Uddback et al. 2024. A group of vaccinated (PR8 SendNP i.n.) and unvaccinated (PR8 WT i.n.) mice were infected and daily measurements of flux using IVIS were performed. A separate group of vaccinated or unvaccinated mice were infected and daily measurements of PFU/mL were performed using the nasal wash plaque assay. Hence, here the measurements for flux and PFU/mL do not come from the same mouse. A linear relationship fitted between the mean $\log_{10} (flux)$and the mean $\log_{10} (PFU/mL)$ is indicated by the black line (adjusted $R^{2}=0.884)$.
