# Supplementary material for: Quantifying the role of pre-existing tissue resident cellular immunity in limiting respiratory virus transmission
Source: PLoS Pathog. 2026 Apr 21;22(4):e1014082. doi: 10.1371/journal.ppat.1014082 (PMC13143178; doi:10.1371/journal.ppat.1014082)
Supplement: S6 Fig — (A) Dynamics of target cells (blue), infected cells (red), viral load (magenta), and interferon (olive green) from the within-host model simulated with’control’ parameters. (B) Dynamics of target cells (blue), infected cells (red), viral load (magenta), and interferon (olive green) from the within-host model simulated with’immune’ parameters. (C) The relationship between simulated infected cell numbers and viral load from the control simulation (A) and immune simulation (B). In the immune group, we see lower infected cell and correspondingly lower viral load compared to the control group. However, the two groups show similar patterns in relating viral load to total infected cell numbers. (D) Diagram of the model showing interactions between different variables. (DOCX) [file ppat.1014082.s006.docx]

**S6 Fig**: **Simulations of within-host viral and immune dynamics. (A)** Dynamics of target cells (blue), infected cells (red), viral load (magenta), and interferon (olive green) from the within-host model simulated with ’control’ parameters. **(B)** Dynamics of target cells (blue), infected cells (red), viral load (magenta), and interferon (olive green) from the within-host model simulated with ’immune’ parameters. **(C)** The relationship between simulated infected cell numbers and viral load from the control simulation (A) and immune simulation (B). In the immune group, we see lower infected cell and correspondingly lower viral load compared to the control group. However, the two groups show similar patterns in relating viral load to total infected cell numbers. **(D)** Diagram of the model showing interactions between different variables**.**
