# Supplementary material for: Quantifying the role of pre-existing tissue resident cellular immunity in limiting respiratory virus transmission
Source: PLoS Pathog. 2026 Apr 21;22(4):e1014082. doi: 10.1371/journal.ppat.1014082 (PMC13143178; doi:10.1371/journal.ppat.1014082)
Supplement: S7 Fig — (A) Comparison between observed and predicted transmission probabilities for the “Short & Fixed” study and (B) “Long & Variable” study. Experimental data was not available for the D5-7 transmission window for the immune group. For the immune group, we used both scontrol(in open circles) and simmune(in open diamonds) to predict transmission probability with simulated immune infection dynamics. This allowed us to study the effect of TRMs on the infection burden alone, keeping infectiousness the same as the control group. As seen in the plots, for both transmission experiments, a reduction in infectiousness for the pre-immune group was necessary to predict the observed transmission probabilities. (DOCX) [file ppat.1014082.s007.docx]

**S7 Fig**: **Model prediction of transmission experiment data. (A)** Comparison between observed and predicted transmission probabilities for the “Short & Fixed” study and **(B)** “Long & Variable” study. Experimental data was not available for the D5-7 transmission window for the immune group. For the immune group, we used both $s_{control}$(in open circles) and $s_{immune}$(in open diamonds) to predict transmission probability with simulated immune infection dynamics. This allowed us to study the effect of TRMs on the infection burden alone, keeping infectiousness the same as the control group. As seen in the plots, for both transmission experiments, a reduction in infectiousness for the pre-immune group was necessary to predict the observed transmission probabilities.
