# Supplementary material for: Quantifying the role of pre-existing tissue resident cellular immunity in limiting respiratory virus transmission
Source: PLoS Pathog. 2026 Apr 21;22(4):e1014082. doi: 10.1371/journal.ppat.1014082 (PMC13143178; doi:10.1371/journal.ppat.1014082)
Supplement: S1 Table — In all the below cases, we fit Ptrans=ea+b×infectionburden1+ea+b×infectionburden. We see that a logistic regression fit with infection burden to be calculated as AUC (log10(flux)) and parameter b being immune-group specific gives the best model fit to the data. (DOCX) [file ppat.1014082.s008.docx]

**S1 Table: Comparison of logistic regression fits to the combined dataset of “Short & Fixed” and “Long & Variable” transmission studies (data presented in Figure 3A).** In all the below cases, we fit $P_{trans}=\frac{e^{a+b\times infection burden}}{1+e^{a+b\times infection burden}}$. We see that a logistic regression fit with infection burden to be calculated as AUC (log10(flux)) and parameter b being immune-group specific gives the best model fit to the data.

| **Logistic regression model form** | **Infection burden calculation** | **AIC** |
| --- | --- | --- |
| Single set of parameters $a$ & $b$ irrespective of index immune status | $AUC({log}_{10} (flux))$ for the transmission window of an index animal | 104.9 |
| Parameter $a$ is immune group specific, while $b$ is constant for both groups |  | 69.3 |
| Parameter $b$ is immune group specific, while $a$ is constant for both groups |  | 66.3 |
| Both parameters $a$ and $b$ are immune group specific |  | 68.3 |
| Single set of parameters $a$ & $b$ irrespective of index immune status | $AUC(flux)$ for the transmission window of an index animal | 72.2 |
| Parameter $a$ is immune group specific, while $b$ is constant for both groups |  | 68.1 |
| Parameter $b$ is immune group specific, while $a$ is constant for both groups |  | 70.9 |
| Both parameters $a$ and $b$ are immune group specific |  | 69.9 |
