# Supplementary material for: Quantifying the role of pre-existing tissue resident cellular immunity in limiting respiratory virus transmission
Source: PLoS Pathog. 2026 Apr 21;22(4):e1014082. doi: 10.1371/journal.ppat.1014082 (PMC13143178; doi:10.1371/journal.ppat.1014082)
Supplement: S2 Table — Below we see that regardless of the form of FOI to model transmission probability, a model with immune-group specific parameters is always favored according to the maximum log-likelihood values. (DOCX) [file ppat.1014082.s009.docx]

**S2 Table: Comparison of FOI forms in explaining the observed data in Figure 3A.** Below we see that regardless of the form of FOI to model transmission probability, a model with immune-group specific parameters is always favored according to the maximum log-likelihood values. In the main text, we report results where FOI was calculated as AUC(log10(flux)) as this functional form best represented the relationship between transmission probability and FOI using the logistic regression method shown in S1 Table.

| **Form of FOI** | **Comments** | **Maximum Log-likelihood value** | **Parameter estimates (SE)** |
| --- | --- | --- | --- |
| $FOI = s\times AUC(log10(flux))$ | Single value of $s$ for both control and immune groups | - 57 | $s=0.1(0.015)$ |
|  | Immune group specific $s$ for control and immune groups | - 40 | $s_{control}=0.19 (0.03)$  $s_{immune}=0.02(0.009)$ |
| $FOI = s\times AUC(flux)$ | Single value of $s$ for both control and immune groups | - 36 | $s=4.9\times{10}^{-9}(2\times{10}^{-5})$ |
|  | Immune group specific $s$ for control and immune groups | - 32.5 | $s_{control}=6.2\times{10}^{-9}(2.9\times{10}^{-5})$  $s_{immune}=1.8\times{10}^{-9}(2.7\times{10}^{-5})$ |
