# Supplementary material for: Quantifying the role of pre-existing tissue resident cellular immunity in limiting respiratory virus transmission
Source: PLoS Pathog. 2026 Apr 21;22(4):e1014082. doi: 10.1371/journal.ppat.1014082 (PMC13143178; doi:10.1371/journal.ppat.1014082)
Supplement: S3 Table — Infection burden in all the below cases is calculated as the AUC(log10(flux)) for an index animal’s transmission window. we see that a logistic regression fit with immune group specific parameters performed equally well to the 2-parameter exponential model fit with immune group-specific parameters. (DOCX) [file ppat.1014082.s010.docx]

**S3 Table: Comparison of different transmission models fitted to the data presented in Figure 3A.** Infection burden in all the below cases is calculated as the AUC(log10(flux)) for an index animal’s transmission window. we see that a logistic regression fit with immune group specific parameters performed equally well to the 2-parameter exponential model fit with immune group-specific parameters. For infectiousness results, we report the analysis done using $n = 1$ and fitting immune-group specific $s$ in the main text, as visually the 2-parameter exponential model looks very similar to the logistic regression model reported in the main text.

| **Model** | **Comments** | **Maximum Log-likelihood value** | **Parameter estimates (SE)** |
| --- | --- | --- | --- |
| Logistic model:  $P_{trans}=\frac{e^{a+b\times infection burden}}{1+e^{a+b\times infection burden}}$ | Single set of parameters $a$ and $b$ for both control and immune groups | - 50 | $a= -3.7 \left( 0.62 \right)$  $b=0.55 (0.1)$ |
|  | Immune group specific parameters $a$ and $b$ for control and immune groups | - 30 | $a_{control}= -6.3 \left( 2.2 \right)$  $b_{control}=1.3 (0.4)$  $a_{immune}= -6.6 \left( 1.9 \right)$  $b_{immune}=0.55 (0.2)$ |
| Exponential model:  $P_{trans}=1-e^{-s\times{infection burden}^{n}}$ | $n=1$;  Single value of $s$ for both control and immune groups | - 57 | $s=0.1(0.015)$ |
|  | $n=1$;  Immune group specific $s$ for control and immune groups | - 40 | $s_{control}=0.19 (0.03)$  $s_{immune}=0.02(0.009)$ |
|  | Single set of parameters $s$ and $n$for both control and immune groups | - 48 | $s=0.01(0.007)$  $n=2.14 (0.3)$ |
|  | Immune group specific $s$ and $n$for control and immune groups | - 31 | $s_{control}= -0.005 \left( 3\times{10}^{-3} \right)$  $n_{control}=3.15 (0.43)$  $s_{immune}= 0.002 \left( {10}^{-3} \right)$  $n_{immune}=2 (0.33)$ |
